# Unraveling the Mystery of COVID-19 Postvaccination Myocarditis: A Systematic Review of Current Cases

**DOI:** 10.1155/2022/2438913

**Published:** 2022-01-31

**Authors:** Hamed Ghoshouni, Sara Bagherieh, Mohammad Parvizinia, Mozhde Askari, Masoumeh Sadeghi, Omid Mirmosayyeb

**Affiliations:** ^1^School of Medicine, Isfahan University of Medical Sciences, Isfahan, Iran; ^2^Cardiac Rehabilitation Research Center, Cardiovascular Research Institute, Isfahan University of Medical Sciences, Isfahan, Iran

## Abstract

From the early stages of the pandemic, the development and mass production of a safe and effective vaccine seemed like the greatest tool, to win the fight against the virus. In the present study, we comprehensively conducted a systematic review of all current cases worldwide to better understand whether there is a link between COVID-19 vaccination and one of the most devastating complications, cardiac Inflammation. Our search retrieved over 250 results, of which 130 met the inclusion criteria, and their respective data were extracted. The results suggest that postvaccination myocarditis and pericarditis are more likely to be seen in male, younger, and mRNA-vaccinated individuals. Most affected patients experienced symptoms following the second shot, and complaint of chest pain was the most prevalent presentation. Currently, no direct link can be drawn between the vaccines and the risk of cardiac inflammation.

## 1. Introduction

Although vaccines have saved hundreds, if not millions, of lives throughout human history, the skepticism surrounding them, especially among the general population, is undeniable [1]. Whether it be normal, anticipated, sporadic side effects temporarily affecting the vaccinated or a major complication assumed to be resulting from the vaccination, antivaxxers have always clung to possible postvaccination complications to disregard the extraordinary efficacy and safety of vaccines [2]. Some of the most controversial complications include the ones affecting the cardiovascular system, as their abrupt and unpredictable nature leaves physicians with a very little amount of time to undo the harm. Myocarditis, pericarditis, and a wide variety of cardiomyopathies are among the most prevalently presumed postvaccination cardiovascular side effects. Also, the COVID-19 vaccine, the most recent and perhaps disputable member of the vaccine's family, is no exception [3, 4].

The very first case of postvaccination myocarditis was reported back in the 1950s, stemming from the smallpox vaccination. Thenceforth, over 200 cases of postvaccination myocarditis and pericarditis have been reported collectively. Nevertheless, the smallpox vaccine was the only type of vaccine to strongly correlate with the findings [5, 6]. Lately, with the ongoing COVID-19 vaccination program around the world, there has been a growing body of research demonstrating cases of postvaccination cardiac complications, highlighting the need for a comprehensive review on present findings to conclude the possible link between COVID-19 vaccination and cardiovascular complications and more specifically, whether or not there is an association between the type of the COVID-19 vaccine and risk of cardiac side effects [7, 8]. Several types of vaccines have been approved by the WHO organization, such as the Pfizer/BioNTech vaccine, the AstraZeneca/AZD1222 vaccine, the Janssen/Ad26.COV 2.S (Johnson & Johnson) vaccine, the Moderna COVID-19 vaccine (mRNA-1273), the Sinopharm COVID-19 vaccine, and the Sinovac-CoronaVac. The number of patients presenting with postvaccination cardiac symptoms varies between the aforementioned types of vaccines, and no studies have evaluated the possible association between the vaccine's type and the risk of cardiovascular complications [9–11].

The present systematic review aims to gather all evidence on the vaccine-related cardiac side effects, in an attempt to provide a more scientific and evidence-based outlook on the possible association between COVID-19 vaccines and subsequent cardiovascular complications.

## 2. Methods

### 2.1. Literature Search

We conducted a systematic computerized search using four data banks: PubMed (MedLine), Scopus, Web of Science, and Embase (via Elsevier). We also searched the gray literature including references from the identified studies, review studies, and conference abstracts which were published up to October 2021.

### 2.2. Inclusion and Exclusion Criteria

Studies reporting any form of postvaccination myocarditis, pericarditis, or cardiomyopathies regardless of the diagnostic tools were included. Nevertheless, articles that were written in any language other than English were excluded.

### 2.3. Data Search and Extraction

We used Mesh terms and text words to generate a syntax that included three components and all their Mesh terms: first, “Myocarditis, Pericarditis, and Cardiomyopathies,” second, “COVID-19,” and third, “vaccines.” Additionally, we customized our search syntax (query) for each data bank not to miss any of the corresponding keywords.

Two researchers independently screened the articles. Any disagreement would be addressed by the senior researcher of the team. The data extraction table included the first author, region of study, date of publication, type of COVID-19 vaccine, age, sex, past medical history, period between vaccination and the onset of cardiac complication, symptoms at presentation, ECG findings, CMR and echocardiography findings, levels of troponin and CRP, COVID-19 diagnostic test, type of cardiac complication, and final treatment and outcome. Had any of the included articles used over one diagnostic method, each different method was mentioned in a separate row of the table with its respective data.

## 3. Findings and Implications

### 3.1. Overview

**Figure 1:**
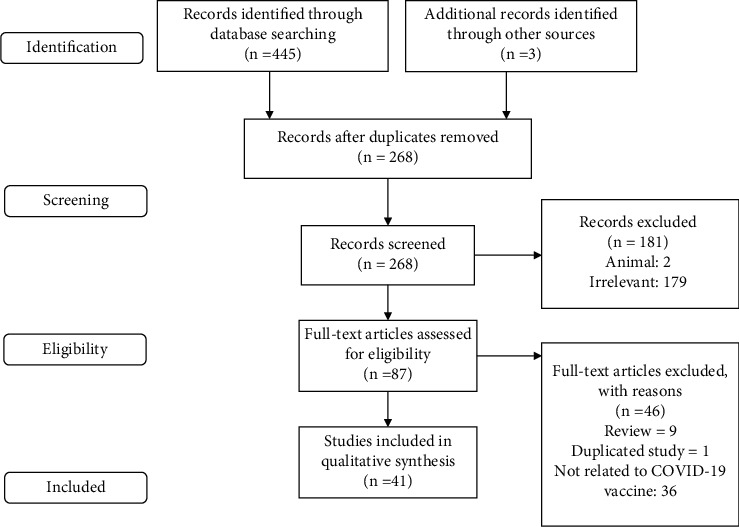
The PRISMA diagram of the included studies.

The literature search found 445 articles. After eliminating duplicates, 268 articles remained, from which 87 full-text articles were included in the final data extraction table. A total of 132 patients were included, 116 males and 16 females, equivalent to 87.88% and 12.12%, respectively (Figure 1). Most of the patients were male and below the age of 30. The male predominance of the patients could be attributed to the variance of sex hormones, as past studies on gender differences observed in inflammatory conditions suggest [12]. About 80% of all patients were vaccinated with mRNA vaccines, including BNT16a2b2 and mRNA-1273. 94.7% of patients experienced symptoms mostly presenting with chest pain. Other less-common symptoms included myalgia, fever, and chills (Supplementary 1). The majority of the following sections will focus on myocarditis-related aspects of postvaccination cardioinflammation due to its heavier health impact and more prevalent nature: however, the same proposed pathophysiology and principles hold true regarding pericarditis.

### 3.2. Critical Appraisal

The quality of all the included articles was assessed using the Joanna Briggs Institute (JBI) critical appraisal checklist. The JBI checklist is the preferred tool for measuring the quality of descriptive studies reporting prevalence data and has a system of ranking articles based on the number of “YES” answers they earn according to its questions. The number of “YES” answers an article can earn ranges between 0 and 9 [13] (Supplementary 2).

### 3.3. The Strategy behind the Inclusion Criteria

The present study is a systematic review of current case reports reporting inflammatory cardiac complications of any sort, with specific respect to myocarditis. It should be noted that the reason behind choosing merely case reports was that we intended to report the detailed characteristics of every patient, which led to the studies designed in any way other than case reports or case series being eliminated from the analysis. As such, any conclusion regarding the prevalence of postvaccination myocarditis should be made carefully based on the data presented in this article, since the number of reported cases in this study might just be the tip of the iceberg.

### 3.4. Timing of Myocarditis

Similar to Rose et al.'s article, our findings suggest that the incidence of new-onset myocarditis following dose 2 was much higher than that of dose 1. Some possible reasons can be contributing to the matter. First, it is safe to assume that underreporting may have happened in regard to the number of myocarditis cases following dose 1 [14], meaning that even if healthy individuals with no underlying health conditions had experienced subclinical levels of cardiac side effects following dose 1, the degree of that would not have been great enough to dissuade them from getting the second dose, leading to the diagnosis of myocarditis being made following the latter. Second, it is noteworthy to mention that the second dose triggers a more immense immune response compared to dose 1, elevating the chances of the vaccinated experiencing cardiac side effects [15]. Third, it is logical to infer those patients whose myocarditis was confirmed following the second dose stood a higher chance of being referred to the research teams of the reviewed articles, implying that, in most of the reviewed cases, follow-up surveys were conducted more rigorously after the second dose and side effects experienced in the period between the two doses could have been assigned to contributing factors other than the vaccine [3, 16].

### 3.5. Association of Age and the Incidence of Myocarditis

Not only are side effects more likely to occur following the second dose, our study suggests that they are more likely to arise in younger individuals [17]. This is in line with the findings of previous studies, where younger populations were more susceptible to suffer from fulminant inflammatory side effects as a result of their stronger immunological response compared to the senile [18, 19]. Besides, it has been shown in past studies that prior exposure to viral products could bring about an amplified mRNA uptake and subsequent immunological response in younger individuals who have recovered from COVID-19 previously, further reinforcing the findings [20]. Nonetheless, it could be deduced that the data released on the elderly might have had some sort of bias, especially in favor of underreporting. In other words, milder forms of myocarditis in elder patients would have easily been overlooked by their possible underlying cardiac diseases [21, 22].

### 3.6. Effects of Vaccine Type on the Incidence of Myocarditis: Potential Mechanisms of Vaccine-Associated Myocarditis

Another aspect of the present study is it demonstrated that the incidence of postvaccination is much higher among people vaccinated with vaccines that use mRNA platforms. The exact reason behind this is still unknown; however, it is noteworthy to mention that one possible scenario is that follow-up surveys were conducted relatively more attentively in mRNA-vaccinated populations [23]. Nevertheless, it is appropriate to add that a maximum observation period of 6 months was the point of reference for approving safety during phase III of mRNA vaccines' clinical trials. This amount of time might need some restructuring, specifically, because this is the very first time mRNA vaccines are being utilized on such a global scale and findings on complications are gathering one case at a time [6, 24].

Besides, mRNA vaccines are nucleoside-modified mRNAs, encoding the viral spike glycoprotein of SARS-CoV-2, and are further encapsulated in lipid nanoparticles. Such vaccines cannot be utilized as direct, unmodified RNA products since that would make them immunogenic and prone to destruction by the native immunity system. Hence, modifications are applied to the selected RNA molecules, reducing their immunogenicity [25]. Once inside the host cells, the mRNA evokes the cell's photosynthesis machinery to produce viral spike proteins and help stimulate adaptive immunity against SARA-CoV-2. Nevertheless, for some genetically predisposed individuals, the modifications have been demonstrated to atypically stimulate the immunologic overreactions rather than attenuating the system. Therefore, in certain vaccinated persons, the proinflammatory cascades and other immunologic pathways including cytokine overexpression may occur following mRNA vaccination [26].

### 3.7. COVID-19-Related Myocarditis

From the early steps of the pandemic, it was recognized that although SARS-CoV-2 was seen as a Coronaviridae family member leading to respiratory illness, it is also capable of causing the multisystem inflammatory syndrome, especially in children and younger adults [7]. Further research elucidated that the virus enters cardiomyocytes employing the angiotensin-converting enzyme 2, an enzyme attached to the membrane of cells in the intestines, kidney, testis, gallbladder, and heart, responsible for lowering blood pressure by catalyzing the hydrolysis of angiotensin II into angiotensin [27]. However, autopsies of patients deceased due to COVID-19 showed a higher number of CD68+ cells, stemming from the monocyte/macrophage linage, as opposed to other commonly known viral causes of myocarditis, in which lymphocytes account for much more diffuse distributions compared to any other immune cell type [28]. Consequently, COVID-19-derived myocarditis is both clinically and histologically different from typical myocarditis observed in other viral processes, as macrophage cells are more capable of involving and fixing the complement system compared to lymphocytes. The activation of the systemic complement system, alongside the local immune system within the heart, leads to the direct death of nearby cardiac cells rather than maintaining them in an inflammatory state for a longer duration similar to other viral diseases [29].

### 3.8. Strengths and Limitations

This study is the first comprehensive systematic review on all the 130 cases of myocarditis and pericarditis following COVID-19 vaccination worldwide. Nevertheless, the present study has some limitations. First, in an attempt to portray a better association between different factors and the risk of postvaccination myocarditis, we omitted all articles but those which had mentioned the detailed characteristics of patients. Second, ECG findings and laboratory data were not analyzed due to the lack of reports in the reviewed articles. This could have been much of help in better realizing the exact characteristics of patients' myocarditis. Notwithstanding all the aforementioned limitations, this study is groundbreaking in that it has reviewed all case report articles worldwide and presented the exact details of the patients.

## 4. Conclusions

In conclusion, postvaccination side effects need close monitoring to gain a more realistic view of possible complications and how best to approach them. The authors, however, would like to herein emphasize the importance of getting vaccinated and advise all individuals to do so as a result of the risk-benefit analysis conducted in the context of experimental studies.

## Data Availability

All of the data will be available for secondary analysis in necessary cases from the corresponding author through e-mail.

## Supplementary Materials

Supplementary Materials Supplementary 1. Overview of all the included studies. Supplementary 2. Quality assessment of all the included studies.
