# Supplementary material for: Unraveling the Mystery of COVID-19 Postvaccination Myocarditis: A Systematic Review of Current Cases
Source: Int J Clin Pract. 2022 Jan 31;2022:2438913. doi: 10.1155/2022/2438913 (PMC9159134; doi:10.1155/2022/2438913)
Supplement: Supplementary Materials — Supplementary 1. Overview of all the included studies. Supplementary 2. Quality assessment of all the included studies. [file 2438913.f1.zip › 2438913.f1/Supp1 (1).docx]

**Supplementary Table 1: This table summarizes key findings of the included studies. Data presented below were used for the purpose of systematic review. (All abbreviations are explained in full terms below the table.)**

| Author | Country | Age  Gender | PMH | COVID vaccine  Dose | Lag between vaccination and presentation | Symptoms at presentation | ECG  ↓: Elevation  ↑: Depression | CMR findings | Echo findings | Lab Data | Treatment | Other Data | Cardiovascular  Adverse Event |
| --- | --- | --- | --- | --- | --- | --- | --- | --- | --- | --- | --- | --- | --- |
| John B. Dickey | USA | 35-40  M  (1) | None | Pf  2nd | 4 d | Chest pain, Neck pain, Chills, Myalgias | I, II, III, aVL, aVF, V5, V6: ST ↑ | LGE | LVEF 45% | Trop + | NA |  | Myocarditis |
| John B. Dickey | USA | 16-20  M  **(1)** | None | Pf  2nd | 3 d | Fever, Chest pain, Rhinorrhea, Headache | Diffuse ST ↑ | LGE | LVEF 53% | Trop + | NA |  | Myocarditis |
| John B. Dickey | USA | 20-25  M  **(1)** | None | Mod  2nd | 4 d | Chest pain, Chills, Myalgias, Fever | Diffuse ST ↑ | LGE | LVEF 58% | Trop + | NA |  | Myocarditis |
| John B. Dickey | USA | 20-25  M  **(1)** | None | Pf  2nd | 2 d | Fever, Chest pain, Myalgia, Malaise | Diffuse ST ↑  PR ↓ | NA | LVEF 48% | Trop + | NA |  | Myocarditis |
| John B. Dickey | USA | 16-20  M  **(1)** | None | Pf  2nd | 4 d | Chest pain, Headache | NL | LGE | LVEF 46% | Trop + | NA |  | Myocarditis |
| John B. Dickey | USA | 16-20  M  **(1)** | None | Pf  2nd | 3 d | Chest pain, Myalgias | Diffuse ST ↑  PR ↓ | NA | LVEF 50% | Trop + | NA |  | Myocarditis |
| Anasua Deb | USA | 67  M  (2) | HTN, DM II, HLP, CAD, CHF, COPD, hypothyroidism, GERD  PCH- | Mod  2nd | 6 h | Fever, Chills, Nausea, Orthopnea, Fatigue, Dyspnea | Sinus tachycardia | NA | LVEF 50-54%  hypokinesia | Trop +  CRP +  ESR +  Pro-BNP + | Diuretics, Bronchodilators, Vasodilators, Antibiotics |  | Myocarditis |
| Joseph Mansour | USA | 25  M  (3) | None | Mod  2nd | 1 d | Fever, Chills, Chest pain | Diffuse ST ↑ | LGE | LVEF 55% | Trop +  CRP +  ESR + | NA |  | Myocarditis |
| Joseph Mansour | USA | 21  F  (3) | None | Mod  2nd | 2 d | Chest pain, Lightheadedness | Diffuse ST ↑  PR ↓ | LGE | LVEF 50% | Trop +  CRP NL  ESR NL  D-Dimer + | NA |  | Myocarditis |
| Javier Bautista Garcı´a | Spain | 39  M  (4) | Asthma, autoimmune hypothyroidism, chronic atrophic gastritis, AF, pneumothorax  PCH- | Pf  2nd | 6 h | Fever, Chest pain | Sinus tachycardia  Diffuse ST ↑  Narrow QRS complex | NA | NL LVEF | Trop + | NA |  | Myocarditis |
| Alberto Cereda | Italy | 21  M  (5) | None | Pf  2nd | 30 h | Fever, Chest pain | Diffuse ST ↑ | LGE  Edema | NL LVEF  hypokinesia | Trop +  CRP + | Antibiotics,β-blocker, ACEI |  | Myocarditis |
| Alon Nevet | Israel | 20  M  (6) | None | Pf  2nd | 2 d | Fever, Chest pain | Diffuse ST ↑ | LGE  Edema | NL LVEF | NA | Colchicine, NSAID |  | Myocarditis |
| Alon Nevet | Israel | 29  M  (6) | None | Pf  2nd | 2 d | Fever, Chest pain | Diffuse ST ↑ | LGE  Edema | NL LVEF | NA | Colchicine, NSAID |  | Myocarditis |
| Alon Nevet | Israel | 24  M  (6) | None | Pf  2nd | 2 d | Fever, Chest pain | Diffuse ST ↑ | LGE  Edema | NL LVEF | NA | Colchicine, NSAID |  | Myocarditis |
| Mhd Baraa Habib | Qatar | 37  M  (7) | HTN | Pf  2nd | 3 d | Chest pain, Body aches, Fever, Chills, Headache | V3-V4: ST ↑ | LGE | LVEF 57 % | Trop + | Antiplatelets, Anticoagulant, β-blocker |  | Myocarditis |
| Anna Patrignani | Italy | 56  M  (8) | PCH + | Pf  1st | 4d | Epigastric pain, Sweating | NL | LGE  Edema | NL LVEF | Trop +  CRP NL  D-Dimer NL | NA |  | Myocarditis |
| Curtis B Williams | Canada | 34  M  (9) | None | Mod  2nd | 1 d | Fevers, Myalgias, Chest pain | I, aVL, V4-V6 : ST ↑  aVR: PR ↑, ST ↓ | LGE  Edema | LVEF 43% | Trop +  CRP +  Pro-BNP + | Antiplatelet, Colchicine, β-blocker, ACEI | CXR: mild pulmonary edema | Myocarditis |
| Dirk Vollmann | Germany | 28  M  (10) | NA | Pf  1st | 9 d | Chest pain, Fatigue, Fever | ST ↑ | LGE  Edema | NA | Trop +  CRP + | NSAID |  | Perimyocarditis |
| In-Cheol Kim | Korea | 24  M  (11) | None | Pf  2nd | 1 d | Chest pain, Myalgia, Fatigue | I, II, aVF, V2-6: ST ↑ | LGE | PE+ | Trop +  CRP +  Pro-BNP + | Symptomatic therapy |  | Myocarditis |
| Yash R. Patel | USA | 22  M  (12) | ADHD | Pf  1st | 2 d | Chest pain, Headache, Malaise, Dyspnea | Diffuse PR ↓  aVR: PR ↑ | LGE  Edema | LVEF 55% | Trop +  CRP + | Antiplatelet, Colchicine |  | Perimyocarditis |
| Yash R. Patel | USA | 19  M  (12) | Asthma | Pf  2nd | 1 d | Chest pain, Dyspnea, Nausea, Vomiting, Emesis | Sinus tachycardia | LGE | LVEF 62% | Trop +  CRP + | Colchicine, NSAID |  | Myocarditis |
| Yash R. Patel | USA | 25  M  (12) | None | Mod  2nd | 3 d | Chest pain, Dyspnea, Body aches, Nausea, Headache, Chills, Fatigue | Diffuse PR ↓  aVR: PR ↑ | LGE  Edema | LVEF 60% | Trop +  CRP + | Colchicine |  | Perimyocarditis |
| Yash R. Patel | USA | 37  M  (12) | None | Pf  2nd | 2 d | Chest pain, Fever, Diaphoresis, Rigors, Nausea, Myalgia, Headache, Fatigue | V5, V6, I , aVL: ST ↑  V1: ST ↓ | LGE  Edema | LVEF 65% | Trop + | NA |  | Myocarditis |
| Yash R. Patel | USA | 20  M  (12) | None | Pf  2nd | 3 d | Chest pain, Dyspnea, Headache, Body ache, Dyspnea | Diffuse PR ↓  aVR : PR ↑ | LGE  Edema | LVEF 51% | Trop + | Colchicine, NSAID, ACEI, β-blocker |  | Perimyocarditis |
| Prashant D. Tailor | USA | 44  M  (13) | obstructive sleep apnoea, asthma , obesity | Mod  2nd | 4 d | Chest pain, Dyspnea, Malaise, Myalgias, Headache, Dry cough | aVL, I , aVR, V1-V6: ST ↑ | LGE  Edema | LVEF 40–45%  hypokinesia | Trop +  CRP +  Pro-BNP + | Diuretics, ACEI, β-blocker, Colchicine | CXR : mild pulmonary edema  Coronary angiography : minimal coronary artery disease | Myocarditis |
| Balraj Singh | USA | 24  M  (14) | None | Pf  2nd | 3 d | Chest pain, Headache, Fever, Chills, Fatigue, Lower back pain | III : ST ↓ | LGE | LVEF 55% | Trop +  CRP +  D-Dimer NL  Pro-BNP NL | NA |  | Myocarditis |
| Mahmoud Nassar | USA | *70*  *F*  (15) | MS  Mahmoud Nassar | J&J  1st | 2 d | Dyspnea, Respiratory distress | Sinus tachycardia, V4-V6: T-wave inversions | NA | LVEF 10%  hypokinesia | Trop +  CRP + | Vasopressors, Antibiotic |  | Myocarditis |
| Antonio Abbate | USA | *27*  *M*  (16) | trisomy 21 | Pf  2nd | 2 d | Nausea, Vomiting | Tachycardia  Diffuse ST ↑ | NA | LVEF 20%  PE+ | CRP +  D-Dimer + | Corticosteroids, IVIG, Anakinra |  | Myocarditis |
| Antonio Abbate | USA | 34  F  (16) | None | Pf  1st | 9 d | Fevers, Cough, Chest pain, Nausea, Vomiting | Tachycardia | LGE | LVEF 15% | CRP +  D-Dimer + | Corticosteroids, IVIG, Anakinra | endomyocardial biopsy:  cytoplasmic vacuolization and lymphocytic infiltrate within cardiomyocytes | Myocarditis |
| Alagarraju Muthukumar | USA | 52  M  (17) | HTN, HLP, obstructive sleep apnea  PCH- | Mod  2nd | 3 d | Chest pain, Fevers, Chills, Myalgias, Headache | Left axis deviation Incomplete RBBB | LGE | NL LVEF | Trop +  CRP +  ESR +  D-Dimer +  Pro-BNP NL | ACEI, β-blocker | Coronary angiography : mild nonobstructive coronary artery disease | Myocarditis |
| Francisco Ujueta | USA | *62*  *F*  (18) | melanoma | J&J  1st | 4 d | Body aches, Weakness, Fatigue | Sinus tachycardia  V1 , V2 :T wave inversions | NA | LVEF 29%  PE+ | Trop +  CRP +  ESR NL  Pro-BNP + | Vasopressin, Phenylephrine, α-agonist, Corticosteroids | Coronary angiography : left ventricular end-diastolic  pressure: 37 mmHg,  pulmonary  artery pressure: 36/30 mm Hg | Myocarditis |
| Kathryn F. Larson | USA | 22  M  (19) | None | Mod  2nd | 3 d | Fever, Chills, Myalgia, Chest pain | Diffuse ST ↑  aVR: ST ↓ | LGE | LVEF 50%  hypokinesia | Trop +  CRP + | NSAID, Corticosteroids |  | Myocarditis |
| Kathryn F. Larson | USA | 31  M  **(19)** | None | Mod  2nd | 3 d | Fever, Chills, Myalgia, Chest pain, Dyspnea | NL | LGE | LVEF 34%  hypokinesia | Trop +  CRP + | No Treatment |  | Myocarditis |
| Kathryn F. Larson | USA | 40  M  (19) | PCH + | Pf  1st | 2 d | Chest pain | Diffuse ST ↑  aVR, V1: ST ↓ | LGE  Edema | LVEF 47%  hypokinesia | Trop +  CRP + | Corticosteroids, Colchicine |  | Myocarditis |
| Kathryn F. Larson | Italy | 56  M  (19) | PCH + | Pf  2nd | 3d | Chest pain | Diffuse peaked T waves | LGE  Edema | LVEF 60%  hypokinesia | Trop +  CRP + | No Treatment |  | Myocarditis |
| Kathryn F. Larson | Italy | 26  M  (19) | None | Pf  2nd | 3 d | Cough, Fever, Chest pain | I,II,III,aVL,aVF, V5, V6; ST ↑ | LGE  Edema | LVEF 60%,  hypokinesia | Trop +  CRP NL | Colchicine |  | Myocarditis |
| Kathryn F. Larson | Italy | 35  M  (19) | None | Pf  2nd | 2 d | Fever, Chest pain | Diffuse ST ↑  aVR: ST ↓ | LGE  Edema | LVEF 50%  hypokinesia | Trop +  CRP + | NSAID |  | Myocarditis |
| Kathryn F. Larson | Italy | 21  M  (19) | None | Pf  2nd | 4 d | Fever, Chest pain | Diffuse ST ↑ | LGE  Edema | LVEF 54%  hypokinesia | Trop +  CRP + | NSAID |  | Myocarditis |
| Kathryn F. Larson | USA | 22  M  (19) | None | Mod  2nd | 2 d | Chest pain | I, II, III, aVF, aVL, V3-V6: ST↑ | LGE  Edema | LVEF 53%  hypokinesia | Trop +  CRP + | No Treatment |  | Myocarditis |
| Kevin Watkins | USA | 20  M  (20) | PCH + | Pf  2nd | 2 d | Chest pain, Dyspnea | Diffuse ST ↑  PR ↓ | Abnormal | LVEF 59% | Trop + | Colchicine, β-blocker, NSAID |  | Myocarditis |
| Tommaso D’ANGELO | ITALY | 30  M  (21) | NA | Pf  2nd | 72 h | Dyspnea, Chest pain, Nausea, Sweating | Tachycardia  V2-V4: ST ↑  V5, V6: nonspecific T-wave changes | LGE | NL LVEF  PE+ | Trop +  CRP + | β-blocker, Antiplatelet, Corticosteroids |  | Perimyocarditis |
| Mahesh K. Vidula | USA | 19  M  (22) | None | Pf  2nd | 4 d | Chest pain, Dyspnea | Diffuse ST ↑ | LGE | LVEF 47% | Trop +  CRP +  ESR + | ACEI, β-blocker |  | Myocarditis |
| Mahesh K. Vidula | USA | 18  M  (22) | None | Mod  2nd | 1 d | Fevers, Myalgias, Chest pain | Diffuse ST ↑ | LGE | NL LVEF | Trop +  CRP + | β-blocker, Colchicine, NSAID |  | Myocarditis |
| Mahesh K. Vidula | USA | 60  F  (22) | IHD | Pf  2nd | 4 d | Chest pain | I, II, III, aVL, aVF, V5, V6: T wave inversions | NA | LVEF 44%  akinesia | Trop + | β-blocker, ACEI |  | Stress Cardiomyopathy |
| Mahesh K. Vidula | USA | 21  F  (22) | ITP | Pf  1st | 3 W | Chest pain | Sinus tachycardia | NA | LVEF 60%  PE+ | CRP +  ESR NL | Colchicine |  | Pericarditis |
| Mahesh K. Vidula | USA | 61  F  (22) | HTN | Pf  2nd | 4 W | Fevers, Sweats, Chest pain, Palpitations | Atrial fibrillation | NA | LVEF 65%  PE+ | CRP +  ESR + | Colchicine |  | Pericarditis |
| Blake Hudson | USA | 24  M  (23) | None | Pf  2nd | 3 d | Myalgias, Fevers, Chills, Nausea, Vomiting, Chest pain | V5, V6, I aVL: J-point ↑  slightly widened QRS complexes | NA | NL LVEF | Trop +  CRP +  ESR NL  D-Dimer +  Pro-BNP NL | Antiplatelet, Colchicine |  | Perimyocarditis |
| Blake Hudson | USA | 22  M  (23) | None | Pf  2nd | 12 h | Chills, Fevers, Chest pain | NL | NA | NL LVEF | Trop +  CRP +  ESR NL  D-Dimer NL | Antiplatelet, Colchicine, NSAID |  | Perimyocarditis |
| Saif Abu Mouch | Israel | 24  M  (24) | None | Pf  2nd | 72 h | Chest pain | Diffuse ST ↑  III : Inverted T | LGE  Edema | NL LVEF | Trop +  CRP + | NA |  | Myocarditis |
| Saif Abu Mouch | Israel | 20  M  (24) | None | Pf  2nd | 24 h | Chest pain | Sinus tachycardia  V2-V6: ST ↑ | LGE  Edema | LVEF 50-55%  hypokinesia | Trop +  CRP + | NA |  | Myocarditis |
| Saif Abu Mouch | Israel | 29  M  (24) | None | Pf  2nd | 48 h | Chest pain | Diffuse PR ↓  Diffuse ST ↑ | LGE  Edema | NL LVEF | Trop +  CRP + | NA |  | Myocarditis |
| Saif Abu Mouch | Israel | 45  M  (24) | None | Pf  2nd | 16 d | Chest pain | I, aVL, V3-5: ST ↑  III, aVF : Inverted T, ST ↓ | LGE  Edema | LVEF 50-55% | Trop +  CRP + | NA |  | Myocarditis |
| Saif Abu Mouch | Israel | 16  M  (24) | None | Pf  2nd | 24 h | Chest pain | V2-4: ST ↑ | LGE  Edema | NL LVEF | Trop +  CRP + | NA |  | Myocarditis |
| Saif Abu Mouch | Israel | 17  M  (24) | None | Pf  2nd | 72 h | Chest pain | I, II, aVL : ST ↑  V2-6 : SI QIII TIII | LGE  Edema | NL LVEF | Trop +  CRP + | NA |  | Perimyocarditis |
| Elisabeth Albert | USA | 24  M  (25) | None | Mod  2nd | 4 d | Chest pain, Fevers, Chills, Body aches | NL | LGE  Edema | LVEF 65% | Trop +  CRP + | β-blocker | CT angiography : small bilateral pleural effusions | Myocarditis |
| William W. King | USA | 23  F  (26) | NA | Mod  2nd | 5 d | Chest pain | PR ↓  Diffuse ST ↑ | LGE | LVEF 55–60%  hypokinesia | Trop +  CRP + | NA |  | Myocarditis |
| William W. King | USA | 20  M  (26) | NA | Mod  2nd | 2 d | Chest pain | PR ↓  Diffuse ST ↑ | NA | LVEF 45%  hypokinesia | Trop +  CRP + | NA |  | Myocarditis |
| William W. King | USA | 29  M  (26) | NA | Mod  2nd | 4 d | Chest pain | Diffuse ST ↑ | NA | LVEF 55% | Trop +  CRP + | NA |  | Myocarditis |
| William W. King | USA | 30  M  (26) | NA | Pf  2nd | 4 d | Chest pain | V5, V6, I aVL: T-wave inversions | NA | LVEF 65–70% | Trop +  CRP + | NA |  | Myocarditis |
| Carolyn M. Rosner | USA | 28  M  (27) | None | J&J  1st | 5 d | Chest pain | II, V5–V6: ST ↑ | LGE | LVEF 51%  hypokinesia | Trop +  CRP NL | β-blocker, ACEI, Antiplatelet |  | Myocarditis |
| Carolyn M. Rosner | USA | 39  M  (27) | None | Pf  2nd | 3 d | Chest pain  Dyspnea | II, aVF, V4–V6: PR ↓  V1: T wave inversion | LGE | LVEF  35-40%  hypokinesia | Trop +  CRP +  Pro-BNP + | β-blocker, ARB, Statin |  | Myocarditis |
| Carolyn M. Rosner | USA | 39  M  (27) | None | Mod  2nd | 4 d | Fever, Chills, Dyspnea, Chest pain | NL | LGE  Edema | LVEF 61% | Trop +  CRP +  Pro-BNP + | Corticosteroids | Coronary angiography :  30% stenosis in proximal circumflex | Myocarditis |
| Carolyn M. Rosner | USA | 24  M  (27) | None | Pf  1st | 7 d | Chest pain, Arm numbness, Tingling | NL | LGE  Edema | LVEF 53% | Trop +  CRP NL  Pro-BNP NL | Colchicine, NSAID, H_2_ blocker |  | Myocarditis |
| Carolyn M. Rosner | USA | 19  M  (27) | None | Pf  2nd | 2 d | Chest pain | NL | LGE  Edema | LVEF 55% | Trop +  CRP +  Pro-BNP + | Colchicine, NSAID, H_2_ blocker |  | Myocarditis |
| Carolyn M. Rosner | USA | 20  M  (27) | None | Pf  2nd | 3 d | Chest pain | V2–V5: ST ↑  Sinus tachycardia | LGE  Edema | LVEF 50%-55%  hypokinesia | Trop +  CRP +  Pro-BNP + | NSAID, H_2_ blocker |  | Myocarditis |
| Carolyn M. Rosner | USA | 23  M  **(27)** | None | Pf  2nd | 3 d | Fevers, Myalgia, Headache, Chest pain | Sinus tachycardia  Diffuse ST ↑ | LGE | LVEF 58% | CRP +  Pro-BNP + | β-blocker, colchicine |  | Myocarditis |
| Bibhuti B Das | USA | 15  M  (28) | None | Pf  2nd | 2 d | Chest pain, Fever, Nausea, Vomiting, Dyspnea | ST ↑ | LGE | LVEF 49% | Trop +  CRP + | NSAID |  | Perimyocarditis |
| Bibhuti B Das | USA | 15  F  (28) | None | Pf  2nd | 2 d | Chest pain | V5, V6, I, aVL: ST↑  T wave inversion | LGE  Edema | NL LVEF | Trop +  CRP + | NSAID |  | Perimyocarditis |
| Bibhuti B Das | USA | 15  M  (28) | None | Pf  2nd | Few  hours | Fatigue, Chills, Dyspnea, Chest pain, Vomiting | V5, V6, I, aVL: T wave inversion  PVC’s | LGE  Edema | NL LVEF | Trop +  CRP + | NSAID |  | Perimyocarditis |
| Bibhuti B Das | USA | 17  M  (28) | None | Pf  1st | 20 d | Chest pain | NL | NA | NL LVEF | Trop + | NSAID |  | Perimyocarditis |
| Bibhuti B Das | USA | 17  M  (28) | None | Pf  2nd | 1 d | Chest pain | ST↑  NS VT | LGE | NL LVEF | Trop +  CRP + | NSAID, IVIG |  | Perimyocarditis |
| Bibhuti B Das | USA | 17  M  (28) | None | Pf  2nd | 4 d | Chest pain | ST↓ | NA | NL LVEF | Trop + | NSAID |  | Perimyocarditis |
| Bibhuti B Das | USA | 16  M  (28) | None | Pf  2nd | 4 d | Chest pain | ST↑  PR↓ | LGE | NL LVEF | Trop +  CRP + | NSAID |  | Perimyocarditis |
| Bibhuti B Das | USA | 15  M  (28) | PCH + | Pf  1st | 3 d | Chest pain | NL | LGE | NL LVEF | Trop +  CRP + | None |  | Perimyocarditis |
| Bibhuti B Das | USA | 16  M  (28) | None | Pf  2nd | 1 d | Chest pain | ST↑ | NA | NL LVEF | Trop + | NSAID |  | Perimyocarditis |
| Bibhuti B Das | USA | 12  M  (28) | None | Pf  2nd | 4 d | Chest pain | ST↑ | NA | NL LVEF | Trop + | NSAID |  | Perimyocarditis |
| Bibhuti B Das | USA | 15  M  (28) | None | Pf  2nd | 2 d | Chest pain | NL | NA | NL LVEF | Trop + | NSAID |  | Perimyocarditis |
| Bibhuti B Das | USA | 15  M  (28) | None | Pf  2nd | 3 d | Chest pain | ST↑ | NA | NL LVEF | Trop + | NSAID |  | Perimyocarditis |
| Bibhuti B Das | USA | 17  M  (28) | None | Pf  2nd | 1 d | Chest pain | ST↑ | NA | NL LVEF | Trop + | NSAID |  | Perimyocarditis |
| Bibhuti B Das | USA | 17  M  (28) | None | Pf  2nd | 2 d | Chest pain, Fever, Chills | ST↑ | LGE | NL LVEF | Trop +  CRP + | NSAID |  | Perimyocarditis |
| Bibhuti B Das | USA | 14  M  (28) | None | Pf  2nd | 2 d | Chest pain, Dyspnea, Shoulder and neck pain | ST↑ | LGE | NL LVEF | Trop +  CRP + | NSAID |  | Perimyocarditis |
| Bibhuti B Das | USA | 14  M  (28) | None | Pf  2nd | 2 d | Chest pain | ST↑ | LGE | NL LVEF | Trop +  CRP + | NSAID |  | Perimyocarditis |
| Bibhuti B Das | USA | 12  M  (28) | None | Pf  2nd | 3 d | Chest pain, Diaphoresis, Tingling of fingers | ST↑ | LGE | NL LVEF | Trop +  CRP + | NSAID |  | Perimyocarditis |
| Bibhuti B Das | USA | 17  M  (28) | None | Pf  2nd | 4 d | Chest pain, Myalgia, Headache | ST↑ | LGE  Edema | NL LVEF | Trop +  CRP + | NSAID |  | Perimyocarditis |
| Bibhuti B Das | USA | 16  M  (28) | None | Pf  2nd | 3 d | Chest pain, Fever, Headache | ST↑ | LGE  Edema | NL LVEF | Trop +  CRP + | NSAID, ACEI, Diuretics |  | Perimyocarditis |
| Bibhuti B Das | USA | 16  F  (28) | None | Pf  1st | 5 d | Chest pain, Syncope | LBBB  Junctional rhythm  first degree AV block  T wave inversion | LGE  Edema | LVEF 48% | Trop +  CRP + | Diuretics, NSAID |  | Perimyocarditis |
| Bibhuti B Das | USA | 13  F  (28) | None | Pf  2nd | 2 d | Chest pain | NL | NL | NL LVEF  PE + | Trop +  CRP + | NSAID |  | Perimyocarditis |
| Bibhuti B Das | USA | 16  M  (28) | None | Pf  2nd | 2 d | Chest pain, Fever | NL | LGE  Edema | NL LVEF | Trop +  CRP + | ACEI, Diuretics |  | Perimyocarditis |
| Bibhuti B Das | USA | 15  M  (28) | None | Pf  2nd | 2 d | Chest Pain | NL | LGE  Edema | NL LVEF | Trop +  CRP + | NSAID |  | Perimyocarditis |
| Bibhuti B Das | USA | 15  M  (28) | None | Pf  2nd | 2 d | Chest pain, Nausea, Vomiting, Fever | Diffuse nonspecific ST changes | LGE  Edema | NL LVEF | Trop +  CRP + | NSAID, IVIG, Corticosteroids |  | Perimyocarditis |
| Bibhuti B Das | USA | 16  M  (28) | None | Pf  2nd | 2 d | Fever, Chest pain | ST ↑ | NA | NL LVEF | Trop +  CRP NL | NSAID |  | Perimyocarditis |
| Kelsey McLean | USA | 16  M  (29) | None | Pf  2nd | 60 h | Chest pain , Myalgias, Fevers | V2 - V6 , I, aVL : diffuse ST ↑ | Abnormal | LVEF 61% | Trop +  CRP +  ESR +  D-Dimer +  Pro-BNP + | IVIG, NSAID |  | Perimyocarditis |
| Alex Fleming-Nouri | USA | 18  M  (30) | None | Pf  2nd | 1 d | Chest pain | II, III, aVF, V3-6: ST↑ | edema,  hyperemia,  fibrosis | NL LVEF | Trop + | NA |  | Perimyocarditis |
| Alex Fleming-Nouri | USA | 21  M  (30) | None | Pf  2nd | 1d | Chest pain | I, II, III, aVF, V4-6: ST↑ | LGE  Edema | NL LVEF | Trop + | NA |  | Perimyocarditis |
| Alex Fleming-Nouri | USA | 17  M  (30) | None | Pf  2nd | 7 d | Chest pain | I, V3-6: ST↑ | LGE | NL LVEF | Trop + | NA |  | Perimyocarditis |
| Alex Fleming-Nouri | USA | 23  M  (30) | None | Pf  2nd | 1 d | Chest pain | V1-3: ST↑,  III, aVF : ST↓ | NA | NL LVEF | Trop + | NA |  | Perimyocarditis |
| Alex Fleming-Nouri | USA | 24  M  (30) | None | Pf  2nd | 3 d | Chest pain | NL | NA | NL LVEF  hypokinesia | Trop + | NA |  | Perimyocarditis |
| Alex Fleming-Nouri | USA | 19  M  (30) | None | Pf  2nd | 1 d | Chest pain | I, II, III, aVF,  V4-6: ST↑ | NL | NL LVEF | Trop + | NA |  | Perimyocarditis |
| Alex Fleming-Nouri | USA | 23  M  **(30)** | None | Pf  2nd | 2 d | Chest pain | II, V3-5: ST↑ | NA | NL LVEF | Trop + | NA |  | Perimyocarditis |
| Alex Fleming-Nouri | USA | 16  M  (30) | None | Pf  2nd | 2 d | Chest pain | II, III, aVF,  V4-6: ST↑ | LGE  Edema | NL LVEF | Trop + | NA |  | Perimyocarditis |
| HanW. Kim | USA | 36  M  (31) | None | Mod  2nd | 3 d | Chest pain, Dyspnea, Fatigue, Muscle ache, Fever, Chills | Diffuse ST ↑  PR ↓ | LGE | NA | Trop +  CRP +  ESR NL | Colchicine, NSAID |  | Myocarditis |
| HanW. Kim | USA | 23  M  (31) | None | Pf  2nd | 5 d | Chest pain, Dyspnea, Syncope, Presyncope, Fatigue, Muscle ache, Fever, Chills | V5, V6, I, aVL: ST ↑ | LGE | NA | Trop +  CRP +  ESR +  Pro-BNP + | Corticosteroids, Colchicine |  | Myocarditis |
| HanW. Kim | USA | 70  F  (31) | HTN, HLP | Mod  2nd | 1 d | Chest pain, Dyspnea, Diaphoresis | I, aVL, V3-V6 : ST ↑ | LGE | NA | Trop +  Pro-BNP + | NA |  | Myocarditis |
| HanW. Kim | USA | 24  M  (31) | None | Pf  2nd | 2 d | Chest pain, Palpitations, Fatigue, Muscle ache, Fever, Chills, Headache | Diffuse ST ↑  PR ↓ | LGE | NA | Trop +  CRP +  ESR NL  Pro-BNP NL | Colchicine, NSAID |  | Myocarditis |
| Ammar A. Hasnie1 | USA | 22  M  (32) | PCH + | Mod  1st | 3 d | Chest pain, Body aches, Fever | Diffuse ST ↑  V3–V6: PR ↓ | LGE | LVEF 50–55%  hypokinesia | Trop +  Pro-BNP NL | Antiplatelet, Colchicine, β-blocker |  | perimyocarditis |
| Eric Tano | USA | 17  M  (33) | Vitiligo  PCH + | Pf  2nd | 4 d | Chest pain | Abnormal T waves  ST ↓ | NA | LVEF 56% | Trop +  D-Dimer NL | NSAID |  | Perimyocarditis |
| Eric Tano | USA | 16  M  (33) | None | Pf  2nd | 3 d | Chest pain, Palpitations | Diffuse ST ↑  PR ↓ | LGE  Edema | LVEF 55% | Trop +  D-Dimer NL | NSAID, Antibiotics, PPI |  | Perimyocarditis |
| Eric Tano | USA | 17  M  (33) | perimyocarditis  PCH - | Pf  1^st^ and 2^nd^ | 3 d | Chest pain | Diffuse ST ↑  PR ↓ | LGE  Edema | LVEF 58% | Trop + | NSAID, IVIG |  | Perimyocarditis |
| Eric Tano | USA | 15  M  (33) | Marfan syndrome, aortic root dilation  PCH + | Pf  1st | 2 d | Chest pain, Fatigue, Abdominal pain | NL | LGE  Edema | LVEF 57% | Trop +  D-Dimer NL | NA |  | Perimyocarditis |
| Eric Tano | USA | 16  M  (33) | PCH - | Pf  2nd | 1 d | Chest pain, Body aches, Headache | Diffuse ST ↑ | NA | NL LVEF | Trop +  D-Dimer + | None |  | Perimyocarditis |
| Eric Tano | USA | 15  M  (33) | Obesity, insulin resistance, dyslipidemia  PCH - | Pf  2nd | 2 d | Chest pain, Fever, Headache, Loose bowel movement, Cough | NL | NA | LVEF 62% | Trop +  D-Dimer + | None |  | Perimyocarditis |
| Eric Tano | USA | 15  M  (33) | None | Pf  2nd | 3 d | Chest pain | Transient ST ↓ | NA | LVEF 60% | Trop +  D-Dimer NL | NSAID, Antibiotics |  | Perimyocarditis |
| Eric Tano | USA | 17  M  (33) | None | Pf  2nd | 1 d | Chest pain | Diffuse ST ↑ | NA | LVEF 61% | Trop + | NSAID |  | Perimyocarditis |
| Kirsten E. Shaw | USA | 24  M  (34) | None | Pf  2nd | 4 d | Chest pain | NA | LGE  Edema | NA | Trop + | NA |  | Myocarditis |
| Kirsten E. Shaw | USA | 31  F  (34) | PCH + | Mod  1st | 25 d | Chest pain | NA | LGE  Edema | NA | Trop + | NA |  | Myocarditis |
| Kirsten E. Shaw | USA | 16  M  (34) | PCH + | Pf  1st | 4 d | Chest pain | NA | LGE  Edema | NA | Trop + | NA |  | Myocarditis |
| Kirsten E. Shaw | USA | 17  F  (34) | None | Pf  2nd | 2 d | Chest pain | V1-V4 : ST ↑ | LGE  Edema | NA | Trop + | NA |  | Myocarditis |
| Prashant K. Minocha | USA | 17  M  (35) | myocarditis | Pf  2nd | 1 d | Chest pain, Fever, Body aches | Diffuse ST ↑ | LGE | NL LVEF | Trop +  CRP +  ESR NL | NSAID |  | Myocarditis |
| Jihyun Park | USA | 15  M  (36) | None | Pf  1st | 3 d | Chest pain | V5, V6, I aVL: ST ↑  left axis deviation | NA | LVEF 63% | Trop +  CRP +  ESR NL  Pro-BNP + | None |  | Myocarditis |
| Jihyun Park | USA | 16  M  (36) | None | Pf  2nd | 2 d | Chest pain | I,II,III,aVF,aVL : ST ↑  T wave inversion | LGE | LVEF 60% | Trop +  CRP +  ESR +  Pro-BNP + | IVIG |  | Myocarditis |
| Mayme Marshall | USA | 16  M  (37) | None | Pf  2nd | 2 d | Fatigue, Poor appetite, Fever, Chest pain | Atrioventricular  dissociation  ST ↑ | LGE | NL LVEF | Trop +  CRP +  ESR +  Pro-BNP + | IVIG, Corticosteroids, NSAID |  | Myocarditis |
| Mayme Marshall | USA | 19  M  (37) | None | Pf  2nd | 3 d | Chest pain, Myalgias, Fatigue, Weakness, Fevers | Diffuse ST ↑ | LGE | NL LVEF | Trop +  CRP +  ESR NL | NSAID, Colchicine, Antiplatelet |  | Myocarditis |
| Mayme Marshall | USA | 17  M  (37) | None | Pf  2nd | 2 d | Chest pain, Paresthesias | Abnormal T waves  Diffuse ST ↑ | LGE  Edema | trace mitral and aortic valve insufficiency | Trop +  CRP +  ESR NL  Pro-BNP + | NSAID |  | Perimyocarditis |
| Mayme Marshall | USA | 18  M  (37) | None | Pf  2nd | 3 d | Chest pain, Malaise, Arthralgia, Myalgia, Fever | ST ↑ | edema, hyperemia, fibrosis | NL LVEF | Trop +  CRP +  ESR + | IVIG, Corticosteroids, NSAID, Antiplatelet |  | Myocarditis |
| Mayme Marshall | USA | 17  M  (37) | None | Pf  2nd | 3 d | Chest pain, Sore throat, Headache, Dry cough, Body aches, Fever | ST ↑ | LGE | NL LVEF | Trop +  CRP +  ESR +  Pro-BNP + | Antibiotics, IVIG, Corticosteroids, NSAID, Antiplatelet |  | Myocarditis |
| Mayme Marshall | USA | 16  M  (37) | None | Pf  2nd | 3 d | Chest pain, Malaise, Fever | Diffuse ST ↑ | LGE  Edema | NL LVEF | Trop +  CRP +  ESR NL  Pro-BNP + | IVIG, Corticosteroids |  | Myocarditis |
| Mayme Marshall | USA | 14  M  (37) | None | Pf  2nd | 2 d | Chest pain, Dyspnea | ST ↑ | LGE  Edema | LVEF 47% | Trop +  CRP +  ESR NL | NSAID, Diuretics |  | Myocarditis |
| Imran Sulemankhil | USA | 33  M  (38) | Asthma, obstructive sleep apnea | J&J  1st | 2 d | Chest pain, Myalgias, Chills | NL | Edema | NA | Trop +  CRP + | NA |  | Myocarditis |
| Enrico Ammirati | Italy | 56  M  (39) | PCH + | Pf  2nd | 3 d | Chest pain | V1-V6 : ST ↑  peaked T waves | LGE  Edema | NA | Trop +  CRP +  D-Dimer + | NA | Coronary angiography : mild plaque in right coronary artery | Myocarditis |
| Fatima Khogali | Qatar | 29  F  (40) | CKD | Mod  2nd | 10 d | Fever, Fatigue, Myalgia, Headache, Nausea, Vomiting, Diarrhea | Diffuse ST ↑  short PR interval | NA | LVEF 27%  PE+ | Trop +  CRP +  D-Dimer NL  Pro-BNP + | Inotropes, Corticosteroids, Colchicine, Antiplatelet |  | Pericarditis |
| Amanda K. Verma | USA | 45  F  (41) | Overweight | Pf  1st | 10 d | Dyspnea, Dizziness | Tachycardia  V5, V6, I, aVL: ST ↓ | LGE | LVEF 15 - 20%  hypokinesia  PE+ | Trop +  CRP +  ESR + | Inotropes  Diuretics, Corticosteroids, ACEI, β-blocker, Antibiotics | Biopsy: inflammatory cells  Coronary angiography : elevated right- and left-sided filling pressures  cardiac index= 1.66 | Myocarditis |
| Amanda K. Verma | USA | *42*  *M*  (41) | None | Mod  2nd | 14 d | Dyspnea , Chest pain, Fever | Diffuse ST ↑ | NA | LVEF: 15%  hypokinesia  PE+ | Trop +  ESR NL  Pro-BNP + | Antiplatelet, Antiarrhythmic, Colchicine, NSAID, Diuretics, Anticoagulants | Autopsy: biventricular  myocarditis | Myocarditis |

**The studies written in *Italic* indicate the cases who passed away.**

M = Male, F = Female

PCH = Previous Covid-19 history

NA = Not Available

PF = Pfizer-BioNTech COVID-19 vaccine (BNT162b2)

Mod = Moderna vaccine (mRNA-1273)

J&J = Johnson and Johnson

LGE = late gadolinium enhancements

ACEI = Angiotensin-converting enzyme inhibitors

NSAID = non-steroidal anti-inflammatory drug

1. Dickey JB, Albert E, Badr M, Laraja KM, Sena LM, Gerson DS, et al. A Series of Patients with Myocarditis Following SARS-CoV-2 Vaccination with mRNA-1279 and BNT162b2. Cardiovascular Imaging. 2021.

2. Deb A, Abdelmalek J, Iwuji K, Nugent K. Acute myocardial injury following COVID-19 vaccination: a case report and review of current evidence from vaccine adverse events reporting system database. Journal of primary care & community health. 2021;12:21501327211029230.

3. Mansour J, Short R, Bhalla S, Woodard P, Verma A, Robinson X, et al. Acute myocarditis after a second dose of the mRNA COVID-19 vaccine: a report of two cases. Clinical Imaging. 2021.

4. García JB, Ortega PP, Fernández JAB, León AC, Burgos LR, Dorta EC. Miocarditis aguda tras administración de vacuna BNT162b2 contra la COVID-19. Revista Espanola De Cardiologia (English Ed). 2021.

5. Cereda A, Conca C, Barbieri L, Ferrante G, Tumminello G, Lucreziotti S, et al. Acute myocarditis after the second dose of SARS-CoV-2 vaccine: Serendipity or atypical causal relationship? Anatolian Journal of Cardiology. 2021;25(7):522.

6. Nevet A. Acute myocarditis associated with anti-COVID-19 vaccination. Clinical and Experimental Vaccine Research. 2021;10(2):196-7.

7. Habib MB, Hamamyh T, Elyas A, Altermanini M, Elhassan M. Acute myocarditis following administration of BNT162b2 vaccine. IDCases. 2021:e01197.

8. Patrignani A, Schicchi N, Calcagnoli F, Falchetti E, Ciampani N, Argalia G, et al. Acute myocarditis following Comirnaty vaccination in a healthy man with previous SARS-CoV-2 infection. Radiology Case Reports. 2021;16(11):3321-5.

9. Williams CB, Choi J-i, Hosseini F, Roberts J, Ramanathan K, Ong K. Acute Myocarditis Following mRNA-1273 SARS-CoV-2 Vaccination. CJC open. 2021.

10. Vollmann D, Eiffert H, Schuster A. Acute Perimyocarditis Following First Dose of mRNA Vaccine Against COVID-19. Deutsches Ärzteblatt International. 2021;118(31-32):546.

11. Kim I-C, Kim H, Lee HJ, Kim JY, Kim J-Y. Cardiac Imaging of Acute Myocarditis Following COVID-19 mRNA Vaccination. Journal of Korean Medical Science. 2021;36(32).

12. Patel YR, Louis DW, Atalay M, Agarwal S, Shah NR. Cardiovascular magnetic resonance findings in young adult patients with acute myocarditis following mRNA COVID-19 vaccination: a case series. Journal of Cardiovascular Magnetic Resonance. 2021;23(1):1-8.

13. Tailor PD, Feighery AM, El-Sabawi B, Prasad A. Case report: acute myocarditis following the second dose of mRNA-1273 SARS-CoV-2 vaccine. European Heart Journal-Case Reports. 2021;5(8):ytab319.

14. Singh B, Kaur P, Cedeno L, Brahimi T, Patel P, Virk H, et al. COVID-19 mRNA Vaccine and Myocarditis. European Journal of Case Reports in Internal Medicine. 2021.

15. Nassar M, Nso N, Gonzalez C, Lakhdar S, Alshamam M, Elshafey M, et al. COVID-19 vaccine-induced myocarditis: Case report with literature review. Diabetes & metabolic syndrome. 2021;15(5):102205.

16. Abbate A, Gavin J, Madanchi N, Kim C, Shah PR, Klein K, et al. Fulminant myocarditis and systemic hyperinflammation temporally associated with BNT162b2 mRNA COVID-19 vaccination in two patients. International Journal of Cardiology. 2021;340:119-21.

17. Muthukumar A, Narasimhan M, Li Q-Z, Mahimainathan L, Hitto I, Fuda F, et al. In Depth Evaluation of a Case of Presumed Myocarditis Following the Second Dose of COVID-19 mRNA Vaccine. Circulation. 2021.

18. Ujueta F, Azimi R, Lozier MR, Poppiti R, Ciment A. Lymphohistocytic Myocarditis after Ad26. COV2. S viral vector COVID-19 vaccination. International journal of cardiology Heart & vasculature. 2021.

19. Larson KF, Ammirati E, Adler ED, Cooper LT, Hong KN, Saponara G, et al. Myocarditis after BNT162b2 and mRNA-1273 Vaccination. Circulation. 2021.

20. Watkins K, Griffin G, Septaric K, Simon EL. Myocarditis after BNT162b2 vaccination in a healthy male. The American Journal of Emergency Medicine. 2021.

21. D'Angelo T, Cattafi A, Carerj ML, Booz C, Ascenti G, Cicero G, et al. Myocarditis after SARS-CoV-2 Vaccination: A Vaccine-induced Reaction? Canadian Journal of Cardiology. 2021.

22. Vidula MK, Ambrose M, Glassberg H, Chokshi N, Chen T, Ferrari VA, et al. Myocarditis and Other Cardiovascular Complications of the mRNA-Based COVID-19 Vaccines. Cureus. 2021;13(6).

23. Hudson B, Mantooth R, DeLaney M. Myocarditis and pericarditis after vaccination for COVID‐19. Journal of the American College of Emergency Physicians Open. 2021;2(4).

24. Mouch SA, Roguin A, Hellou E, Ishai A, Shoshan U, Mahamid L, et al. Myocarditis following COVID-19 mRNA vaccination. vaccine. 2021.

25. Albert E, Aurigemma G, Saucedo J, Gerson DS. Myocarditis following COVID-19 vaccination. Radiology case reports. 2021;16(8):2142-5.

26. King WW, Petersen MR, Matar RM, Budweg JB, Pardo LC, Petersen JW. Myocarditis following mRNA vaccination against SARS-CoV-2, a case series. American Heart Journal Plus: Cardiology Research and Practice. 2021;8:100042.

27. Rosner CM, Genovese L, Tehrani BN, Atkins M, Bakhshi H, Chaudhri S, et al. Myocarditis Temporally Associated with COVID-19 Vaccination. Circulation. 2021.

28. Das BB, Kohli U, Ramachandran P, Nguyen HH, Greil G, Hussain T, et al. Myopericarditis following mRNA COVID-19 vaccination in adolescents 12 through 18 years of age. The Journal of pediatrics. 2021.

29. McLean K, Johnson TJ. Myopericarditis in a Previously Healthy Adolescent Male Following COVID‐19 Vaccination: A Case Report. Academic Emergency Medicine. 2021.

30. Fleming‐Nouri A, Haimovich AD, Yang D, Schulz WL, Coppi A, Taylor RA. Myopericarditis in young adults presenting to the emergency department after receiving a second COVID‐19 mRNA vaccine. Academic Emergency Medicine. 2021;28(7):802.

31. Kim HW, Jenista ER, Wendell DC, Azevedo CF, Campbell MJ, Darty SN, et al. Patients with acute myocarditis following mRNA COVID-19 vaccination. JAMA cardiology. 2021.

32. Hasnie AA, Hasnie UA, Patel N, Aziz MU, Xie M, Lloyd SG, et al. Perimyocarditis following first dose of the mRNA-1273 SARS-CoV-2 (Moderna) vaccine in a healthy young male: a case report. BMC Cardiovascular Disorders. 2021;21(1):1-6.

33. Tano E, San Martin S, Girgis S, Martinez-Fernandez Y, Vegas CS. Perimyocarditis in adolescents after Pfizer-BioNTech COVID-19 vaccine. Journal of the Pediatric Infectious Diseases Society. 2021.

34. Shaw KE, Cavalcante JL, Han BK, Gössl M. Possible Association Between COVID-19 Vaccine and Myocarditis: Clinical and CMR Findings. Cardiovascular Imaging. 2021.

35. Minocha PK, Better D, Singh RK, Hoque T. Recurrence of acute myocarditis temporally associated with receipt of the mRNA COVID-19 vaccine in an adolescent male. The Journal of Pediatrics. 2021.

36. Park J, Brekke DR, Bratincsak A. Self-limited myocarditis presenting with chest pain and ST segment elevation in adolescents after vaccination with the BNT162b2 mRNA vaccine. Cardiology in the Young. 2021:1-4.

37. Marshall M, Ferguson ID, Lewis P, Jaggi P, Gagliardo C, Collins JS, et al. Symptomatic acute myocarditis in seven adolescents following Pfizer-BioNTech COVID-19 vaccination. Pediatrics. 2021:2.

38. Sulemankhil I, Abdelrahman M, Negi SI. Temporal association between the COVID-19 Ad26. COV2. S vaccine and acute myocarditis: A case report and literature review. Cardiovascular Revascularization Medicine. 2021.

39. Ammirati E, Cavalotti C, Milazzo A, Pedrotti P, Soriano F, Schroeder JW, et al. Temporal relation between second dose BNT162b2 mRNA Covid-19 vaccine and cardiac involvement in a patient with previous SARS-COV-2 infection. International journal of cardiology Heart & vasculature. 2021.

40. Khogali F, Abdelrahman R. Unusual Presentation of Acute Perimyocarditis Following SARS-COV-2 mRNA-1237 Moderna Vaccination. Cureus. 2021;13(7).

41. Verma AK, Lavine KJ, Lin C-Y. Myocarditis after covid-19 mRNA vaccination. New England Journal of Medicine. 2021.
