# Supplementary material for: Unraveling the Mystery of COVID-19 Postvaccination Myocarditis: A Systematic Review of Current Cases
Source: Int J Clin Pract. 2022 Jan 31;2022:2438913. doi: 10.1155/2022/2438913 (PMC9159134; doi:10.1155/2022/2438913)
Supplement: Supplementary Materials — Supplementary 1. Overview of all the included studies. Supplementary 2. Quality assessment of all the included studies. [file 2438913.f1.zip › 2438913.f1/Supp2 (1).docx]

| Author | **1. Were patient’s demographic characteristics clearly described?** | **2. Was the patient’s history clearly described and presented as a timeline?** | **3. Was the current clinical condition of the patient on presentation clearly described?** | **4. Were diagnostic tests or assessment methods and the results clearly described?** | **5. Was the intervention(s) or treatment procedure(s) clearly described?** | **6. Was the post-intervention clinical condition clearly described?** | **7. Were adverse events (harms) or unanticipated events identified and described?** | **8. Does the case report provide takeaway lessons?** | **Score** |
| --- | --- | --- | --- | --- | --- | --- | --- | --- | --- |
| Anasua Deb | Yes | Yes | Yes | Yes | Yes | Yes | Yes | Yes | 8 |
| Javier Bautista Garcı´a | Yes | Yes | Yes | Yes | Unclear | Yes | Yes | Yes | 7 |
| Alberto Cereda | Yes | Yes | Yes | Yes | No | Yes | Yes | Yes | 7 |
| Mhd Baraa Habib | Yes | Yes | Yes | Yes | Yes | Yes | Yes | Yes | 8 |
| Anna Patrignani | Yes | Yes | Yes | Yes | Unclear | Yes | Yes | Yes | 7 |
| Curtis B Williams | Yes | Yes | Yes | Yes | Yes | Yes | Yes | Yes | 8 |
| Dirk Vollmann, | Yes | No | Yes | Yes | Yes | Yes | Yes | Yes | 7 |
| In-Cheol Kim | Yes | Yes | Yes | Yes | Unclear | Yes | Yes | Yes | 7 |
| Prashant D. Tailor | Yes | Yes | Yes | Yes | Yes | Yes | Yes | Yes | 8 |
| Balraj Singh | Yes | Yes | Yes | Yes | Unclear | Yes | Yes | Yes | 7 |
| Mahmoud Nassar | Yes | Yes | Yes | Yes | Yes | Yes | Yes | Yes | 8 |
| Alagarraju Muthukumar | Yes | Yes | Yes | Yes | Yes | Yes | Yes | Yes | 8 |
| Francisco Ujueta | Yes | Yes | Yes | Yes | Yes | Yes | Yes | Yes | 8 |
| Kevin Watkins | Yes | Yes | Yes | Yes | Yes | Yes | Yes | Yes | 8 |
| Tommaso D’ANGELO | Yes | No | Yes | Yes | Yes | Yes | Yes | Yes | 7 |
| Elisabeth Albert | Yes | Yes | Yes | Yes | No | No | Yes | Yes | 6 |
| Kelsey McLean | Yes | Yes | Yes | Yes | Yes | Yes | Yes | Yes | 8 |
| Ammar A. Hasnie1 | Yes | Yes | Yes | Yes | Yes | Yes | Yes | Yes | 8 |
| Prashant K. Minocha | Yes | Yes | Yes | Yes | Yes | Yes | Yes | Yes | 8 |
| Imran Sulemankhil | Yes | Yes | Yes | Yes | Unclear | Yes | Yes | Yes | 7 |
| Enrico Ammirati | Yes | Yes | Yes | Yes | Unclear | Unclear | Yes | Yes | 6 |
| Fatima Khogali | Yes | Yes | Yes | Yes | Yes | Yes | Yes | Yes | 8 |

**Quality Assessment of the included case report studies based on the Joanna Briggs Institute (JBI) critical appraisal checklist.**

**Quality Assessment of the included case series studies based on the Joanna Briggs Institute (JBI) critical appraisal checklist.**

| Author | **1. Were there clear criteria for inclusion in the case series?** | **2. Was the condition measured in a standard, reliable way for all participants included in the case series?** | **3. Were valid methods used for identification of the condition for all participants included in the case series?** | **4. Did the case series have consecutive inclusion of participants?!** | **5. Did the case series have complete inclusion of participants?!** | **6. Was there clear reporting of the demographics of the participants in the study?** | **7. Was there clear reporting of clinical information of the participants?** | **8. Were the outcomes or follow up results of cases clearly reported?** | **9. Was there clear reporting of the presenting site(s)/clinic(s) demographic information?!** | **10. Was statistical analysis appropriate?** | **Score** |
| --- | --- | --- | --- | --- | --- | --- | --- | --- | --- | --- | --- |
| John B. Dickey | Yes | Yes | Yes | No | No | Unclear | Yes | No | No | No | 4 |
| Joseph Mansour | Yes | Yes | Yes | No | Yes | Yes | Yes | Unclear | No | No | 6 |
| Alon Nevet | Yes | Yes | Yes | No | Yes | Yes | Yes | Unclear | No | No | 6 |
| Yash R. Patel | Yes | Yes | Yes | No | Yes | Yes | Yes | Yes | No | No | 7 |
| Antonio Abbate | Yes | Yes | Yes | Yes | Yes | Yes | Yes | Yes | No | No | 8 |
| Kathryn F. Larson | Yes | Yes | Yes | No | Yes | Yes | Yes | Yes | No | No | 7 |
| Mahesh K. Vidula | Yes | Yes | Yes | Yes | Yes | Yes | Yes | Yes | No | No | 8 |
| Blake Hudson | Yes | Yes | Yes | No | Yes | Yes | Yes | Yes | No | No | 7 |
| Saif Abu Mouch | Yes | Yes | Yes | Yes | Yes | Yes | Yes | Yes | No | No | 8 |
| William W. King | Yes | Yes | Yes | No | Yes | Yes | Yes | Yes | No | No | 7 |
| Carolyn M. Rosner | Yes | Yes | Yes | Yes | Yes | Yes | Yes | Yes | Yes | No | 9 |
| Bibhuti B Das | Yes | Yes | Yes | No | Yes | Yes | Yes | Unclear | No | No | 6 |
| Alex Fleming-Nouri | Yes | Yes | Yes | Yes | Yes | Yes | Yes | Unclear | No | No | 7 |
| HanW. Kim | Yes | Yes | Yes | No | Yes | Yes | Yes | Yes | No | No | 7 |
| Eric Tano | Yes | Yes | Yes | Yes | Yes | Yes | Yes | Unclear | No | No | 7 |
| Kirsten E. Shaw | Yes | Yes | Yes | No | Yes | Yes | Yes | No | No | No | 6 |
| Jihyun Park1 | Yes | Yes | Yes | No | Yes | Yes | Yes | Yes | No | No | 7 |
| Mayme Marshall | Yes | Yes | Yes | Yes | Yes | Yes | Yes | Yes | No | No | 8 |
| Amanda K. Verma | Yes | Yes | Yes | No | Yes | Yes | Yes | Yes | No | No | 7 |
